# P-2182. Assessing Hepatitis A Virus (HAV) and Hepatitis B Virus (HBV) Screening, Immunity, and Vaccination in People Living with Human Immunodeficiency Virus (HIV) in the Illinois Department of Corrections (IDOC)

**DOI:** 10.1093/ofid/ofae631.2336

**Published:** 2025-01-29

**Authors:** Nicholas T Truong, Alex Dang, Hillary Debs, Jennifer Morrow, Huda Kalota, Mahesh C Patel, Scott Borgetti, Emily N Drwiega, Melissa E Badowski

**Affiliations:** University of Illinois Chicago, Chicago, Illinois; University of Illinois Chicago, Chicago, Illinois; University of Illinois Chicago, Chicago, Illinois; University of Illinois Chicago, Chicago, Illinois; University of Illinois Chicago, Chicago, Illinois; University of Illinois Chicago, Chicago, Illinois; University of Illinois at Chicago, Chicago, Illinois; University of Illinois Chicago, Chicago, Illinois; University of Illinois Chicago, Chicago, Illinois

## Abstract

**Background:**

Individuals in custody are highly susceptible to vaccine-preventable diseases, including hepatitis A virus (HAV) and hepatitis B virus (HBV). In the Illinois Department of Corrections (IDOC) HIV Telemedicine Clinic, people are screened for HAV/HBV, providing an opportunity to recommend vaccines in those without immunity.

**Figure:**
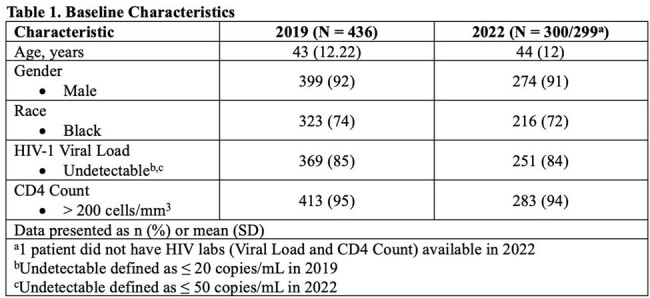

**Methods:**

This retrospective cohort pre-post study included adults living with HIV who followed in the IDOC HIV Telemedicine Clinic during two time periods, 01/01/2019 - 12/31/2019 and 01/01/2022 – 12/31/2022, with a 9-month follow-up period to account for screening and vaccination recommendations. Patients were excluded from primary endpoint analysis if they had chronic HBV infection. The primary objective was to compare the rates of vaccination and immunity to HAV and/or HBV among individuals in custody living with HIV in 2022 to prior data in 2019, where standardized recommendations for serologic screening and vaccination were not routinely performed. Secondary endpoints included the number of individuals receiving HAV/HBV screening, acceptance of vaccination if recommended, and documented barriers to vaccination. Statistical analysis included descriptive statistics, Chi-squared test, and Student’s t-test.

**Figure:**
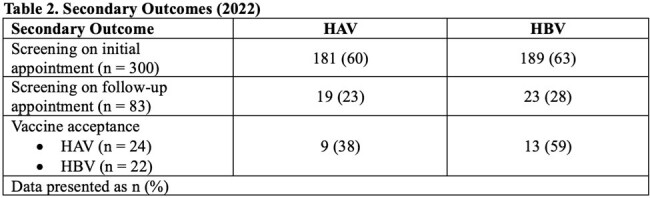

**Results:**

A total of 436 patients were included in the 2019 cohort and served as historical control data. Of 341 patients screened for inclusion in the 2022 cohort, 300 were included. Baseline characteristics are presented in Table 1. 79% and 70% of patients were immune to HAV in 2019 and 2022, respectively (p = 0.02). 65% and 71% of patients were immune to HBV in 2019 and 2022, respectively (p = 0.12). Overall, the number of patients immune to both HAV and HBV were similar between the two cohorts with 52% in 2019 and 53% in 2022 (p = 0.89). On the other hand, the percentage of patients who had no immunity to both HAV and HBV was 8% in 2019 and 12% in 2022 (p = 0.08). Patient refusal was the main barrier to vaccination. Other secondary outcomes for 2022 are outlined in Table 2.

**Conclusion:**

Overall, HAV and HBV immunity did not significantly increase in 2022. Results suggest that a major opportunity still exists to screen and provide vaccination to individuals in custody, but routine serologic screening can lead to improved HAV and HBV immunity rates.

**Disclosures:**

Scott Borgetti, MD, GSK: Grant/Research Support

